# Depopulation

**Published:** 1876-07

**Authors:** 


### DEPOPULATION.

THE Society of Friends, called Quakers, are allowed by common consent to be the most exemplary people in the world; no sect or class exceeds them in integrity, industry, and in individual deportment as to all the proprieties of life. While as a community they are prosperous in business, live in simple comfort and abundance, there is a thrift about them individually, which is the admiration of all who know them. In a million of paupers, or beggars, or criminals, there will scarcely be found a single "Friend;" and yet they are depopulating with a greater rapidity than the Sandwich Islanders, whom wasting diseases decimate every few years. In 1690 there were in Great Britain and Ireland some seventy thousand "Friends;" to-day there are not more than twenty-six thousand, although the population of those countries has been trebled. Since 1810, the deaths among Friends in Great Britain have exceeded the births by twenty-four hundred. These facts strikingly show how statistics may be read amiss, and how actual figures may lie; for at first glance it would seem that they were wasting away by disease, when it would follow that industry, thrift, and a blameless life not only failed to give length of days, but promoted premature decline and death. This would be on a par with the reasoning of some wicked men, who assert that because more than half the Sandwich Islanders have died off within the memory of men, and that within that time they have been civilized and Christianized, that Christianity is the cause of their numerical decline; when the true reason for it is known to be the introduction of the small-pox in part, but in greater part the dissemination of an infamous disease among them by French and English sailors, and by the introduction of brandy at the muzzles of the cannon of French men-of-war. Hence, so far from missionary labor being the cause of their depopulation, it is to missionary labor, its antagonizing influences as to drunkenness, idleness, and effeminacy, the fact is owing that a Sandwich Islander lives to prove that such a people ever existed.

As to the "Friends" in Great Britain, so far from their blameless lives being a cause of their depopulation, it is known that they live longer than the people around them, by an average of fifteen years. Hence a cause of their decline must be looked for elsewhere than in a physical, physiological, or hygienic point of view. Premiums have been offered for the best essays as to the causes of a decline which thinking men contemplate with a melancholy regret. The general opinion, outside of themselves, seems to be that they have declined in numbers from the strictness of their discipline and their dislike of a "hireling ministry." Whatever may be the cause of their decline, that decline, and to their credit be it spoken, is a source of sincere regret by the reflecting and the good.

